# Recurrence of fatty liver disease following liver transplantation for NAFLD-related cirrhosis: Current status and challenges

**DOI:** 10.22088/cjim.11.4.346

**Published:** 2020

**Authors:** Arash Dooghaie Moghadam, Pegah Eslami, Niloofar Razavi-Khorasani, Bobak Moazzami, Farbod Zahedi-Tajrishi, Ermia Farokhi, Kamyab Makhdoomi Sharabiani, Alireza Mansour-Ghanaei, Azim Mehrvar, Morteza Aghajanpoor Pasha, Sandra Saeedi, Shahrokh Iravani

**Affiliations:** 1 Liver Transplantation Research Center, Tehran University of Medical Sciences, Tehran, Iran; 2 Research Institute for Gastroenterology and Liver Diseases, Shahid Beheshti University of Medical Sciences, Tehran, Iran; 3 Student Research Committee, Babol University of Medical Sciences, Babol, Iran; 4 Research Center for Cancer Screening and Epidemiology, AJA University of Medical Sciences, Tehran, Iran; 5 Gastroenterology and Hepatobiliary Research Center, AJA University of Medical Sciences, Tehran, Iran

**Keywords:** Liver transplantation, Non-alcoholic fatty liver disease, Hepatic steatosis; Steatohepatitis, Liver cirrhosis, Metabolic syndrome, Insulin resistance, Obesity

## Abstract

Non-alcoholic fatty liver disease (NAFLD) is emerging as a major health problem worldwide. NAFLD is a continuum of disease ranging from mild liver steatosis to severe steatohepatitis, which will ultimately lead to end-stage liver disease with high morbidity and mortality rates. This disorder is considered as a silent liver disease. The metabolic syndrome and its components are accounted as the major risk factors for the progression of NAFLD to NASH and cirrhosis. Liver transplantation is considered as an appropriate treatment for the end-stage disease. For the last two decades, NASH has been the most common reason for liver transplantation, especially in the developed countries; however, the outcome of post-transplantation in these patients is of a great concern. The recurrent NASH and NAFLD seem to be the usual issues in LT. Steatosis appears in more than 80% of LTs; however, re-transplantation caused by steatohepatitis is rare. Recently, several risk factors of the recurrent NAFLD, including age, donor steatosis, metabolic syndrome, and immunosuppressant agents, have been introduced. Among the metabolic syndrome components, obesity seriously has negative effects on the outcomes of post-liver transplantation in patients. Unfortunately, there is no standard medicine to prevent or treat the recurrent NAFLD; however, it seems that weight loss and lifestyle modification play critical roles in controlling or inhibiting the recurrent NAFLD or NASH.

Non-alcoholic fatty liver disease (NAFLD) is a common chronic liver disease worldwide. This disorder can lead to end-stage liver disease and encompasses a spectrum of liver damages ranging from uncomplicated steatosis to cirrhosis (1). Liver transplantation (LT) has been considered as the treatment of choice for nonalcoholic fatty liver disease (NASH) - related cirrhosis. Moreover, NASH is the most common etiology for LT in industrial countries (2, 3). Although LT is a standard treatment used for the NASH cases, there is limited information on the prevalence of the disorder recurrence in patients undergoing transplantation because of NASH (4). Furthermore, the best prevention or treatment techniques for the recurrent NAFLD are still unknown (5). Accordingly, we evaluated the previous studies on the prevalence ratio of the recurrent NAFLD in the LT patients. Moreover, we spared our efforts to detect the best management methods for decreasing and controlling this disorder that could exacerbate the outcome of liver transplantation. 

**Pathogenesis: **Although multiple mechanisms have been proposed for the pathogenesis of NAFLD, the exact underlying etiology remains yet to be fully elucidated (6). Environmental and genetic factors as well as insulin resistance have been proposed to play a pivotal role in progression to NAFLD (7, 8). The initial accumulation of triglycerides within hepatocytes (i.e., steatosis) triggers the hepatocellular injury (9). This steatosis can be further complicated by death and inflammation of hepatocytes; a condition known as nonalcoholic steatohepatitis (NASH). On the other hand, individuals with genetic variants in the Patatin-like phospholipase domain containing 3 (PNPLA3), responsible for hydrolysis of triacylglycerol molecules in adipocytes, are susceptible to NAFLD development regardless of the existence of metabolic syndrome (10, 11). Moreover, alteration in transmembrane 6 superfamily member 2, encoding E167K (rs58542926 C/T), and lipid transporter located endoplasmic reticulum could lead to the impairment of lipid disposal pathways and resulted in hepatic accumulation of triglycerides (12). Therefore, the three main steps in the progression to NAFLD is steatosis, lipotoxicity and inflammation, collectively known as ‘three-hit’ process (13).

Previous studies showed a strong association between steatosis development and diets, gut microbiota, genetic background, and regulation of de novo lipogenesis via lipogenic transcription factors including sterol regulatory binding protein-1c (SREBP1c) that were involved in mediating the effect of insulin on hepatic gene expression, and carbohydrate-responsive element-binding protein (chREBP) that were regulated by glucose for gene transcription(14-16). The adipose tissue is the primary site for storage of triacylglycerol (TAG) and during periods of energy deprivation, fatty acids generated from TAG hydrolysis will used as a fuel by various organs. However, an ectopic fat deposition in tissues other than adipose tissue seems to occur among the obese subjects. It has been shown that the upregulation of fatty acid transport proteins (FATPs) and FAT/CD36 (fatty acid translocase) that is commonly elevated in obese cases and NAFLD patients, resulted in the increased uptake of fatty acid by organs such as the skeletal and hepatic tissues (17, 18). Excess fatty acid accumulation in the liver resulted in lipotoxicity via amplification of oxidative stress leading to organelle dysfunction, which mainly derived from mitochondrial dysfunction (19, 20). Therefore, the oxidation of free fatty acids by dysfunctional mitochondria leads to the over production of reactive oxygen species (ROS) that is known as the primary cause of oxidative stress (21). Finally, oxidative stress in NAFLD patients (also characterized by the third insult) will ultimately result in hepatocyte death and demonstrated as histopathological alterations and biochemical features of the liver involvement (22). The genetic and epigenetic factors affecting NAFLD are also of great importance. The PNPLA3 gene encodes an enzyme responsible for intracellular trafficking of lipids, hence variants of this gene could play a crucial role in the progression to steatosis as well as the susceptibility to hepatocellular malignancies (23). The genes TM6SF2 (24) and MBOAT7 (25) are also involved in lipid homeostasis, making them important factors in the development of steatosis as well.

**Epidemiology: **The global incidence of NAFLD has increased significantly over the past few decades (table 1) (26). A cohort conducted in the United States reported that nearly 33% of Americans were diagnosed with NAFLD, although the reported diagnoses of NAFLD were not validated by histological studies (27, 28). According to the International Liver Transplantation Society (ILTS) consensus conference on NAFLD and liver transplantation, the prevalence rate of NAFLD is about 25% worldwide and it became the most common chronic liver disease. In addition, they reported a significant direct correlation between obesity and the incidence of NAFLD (28). Differences in sex and race/ethnicity can affect the prevalence rate of NAFLD. For instance, NAFLD was shown to be more prevalent in Hispanics when compared to African-Americans in several studies (29, 30). This fact could partly be explained by different lifestyles and body fat distributions among the different ethnic groups. The increased incidence of NAFLD is also frequently found among the potential liver transplant donors. In many occasions, liver studies of a seemingly healthy candidate donor reveal that the donor has already been affected by the terminal stage of the liver disease (31).

**Liver transplantation: **LT is a surgical treatment technique for all types of end-stage hepatic disease. Although it is frequently used as the treatment of hepatitis C, LT is now being investigated as the treatment of NAFLD (32, 33). Such increasing trend can be explained by global rapid lifestyle changes towards an unhealthy and sedentary one, which promotes obesity, hypertension, diabetes, and hepatitis among many other etiologies. With the recent improvements in non-surgical methods used to treat viral hepatitis, the LT would predominantly be used to treat NAFLD instead of viral hepatitis in the near future. The 1, 3 and 5 year survival rates of LT patients diagnosed with NASH are 87.6%, 82.2% and 76.7%, respectively (21). Nevertheless, LT alone does not guarantee the beneficial long-term outcome. Post- transplantation treatment techniques are also of great importance as they severely affect the prognosis of surgery. Accordingly, although the new liver may result in the patients’ improved overall function, it is at an increased risk of developing recurrent NAFLD or de novo form since the patient’s lifestyle is not improved (34). 

**Table 1:** An overview of current studies on NAFLD recurrence following liver transplant

**Author **	**Year**	**Title**	**Aim**	**Sample**	**Conclusion**
Achuthan Sourianarayanane(4)	2017	Nonalcoholic steatohepatitis recurrence and rate of fibrosis progression following liver transplantation	The comparison of incidences of NASH following LT for NASH with those transplanted for alcoholic liver disease (ALD).	NASH patients = 77 ALD patients= 108	More steatosis and inflammation in NASH arm. progression of fibrosis was more rapid in ALD Arm.
Rohan C. Siriwardana(75)	2015	Recurrence of graft steatosis after liver transplantation for cryptogenic cirrhosis in recently commenced liver transplant program	The short-term follow-up of graft histology in LT patients with previous NAFLD.	Five patients, with cryptogenic cirrhosis underwent liver transplantation from 2012 to 2013	High prevalence of graft steatosis due to recurrent NAFLD was detected.
M_elanie Vallin(41)	2014	Recurrent or de novo Nonalcoholic Fatty Liver Disease After Liver Transplantation: Natural History Based on Liver Biopsy Analysis	Comparison the different aspects of recurrent NAFLD and de novo forms in a cohort study, during 5 years of follow up	De novo arm = 80 Recurrent arm = 11	Recurrent NAFLD was more severe, and irreversible.
El Atrache MM(76)	2012	Recurrence of non-alcoholic steatohepatitis and cryptogenic cirrhosis following orthotopic liver transplantation in the context of the metabolic syndrome	Determination of the prevalence of recurrent NAFLD/NASH in LT patients with previous NASH diagnosis.	83	Metabolic syndrome has an important role in recurrent NASH in post-transplant patients.
Parul Dureja(37)	2011	NAFLD Recurrence in Liver Transplant Recipients	Evaluation of the prevalence of recurrent NAFLD in post-transplant patients in a cohort study between 1993-2007	88	1. Recurrent NAFLD is common in the 5 years. Significant relation between Metabolic syndrome and recurrent NAFLD. 3. No relationship with high mortality.

**The definition of post liver transplant-related de novo vs recurrent NAFLD: **Post-op NAFLD can be categorized into two distinct subgroups: recurrent and de novo (35). Recurrent NAFLD is defined by the development of fatty liver following LT in the context of a previous NAFLD (35). In other words, such patients undergo LT surgery because of severe steatohepatitis or cirrhosis. As previously addressed, the underlying etiology of such diseases is a steatotic liver. De novo NAFLD indicates that the steatosis is a new pathology, occurring in a patient with previously diagnosed cirrhosis or hepatitis of different etiologies (e.g alcoholic or viral hepatitis) (36). The subgroups in these two categories are different in etiology, prevalence and to a lesser extent and management. 

**Prevalence of NAFLD among post-liver transplant patients: **There are multiple reports on this matter, mostly assessing the rate of steatosis, NAFLD, steatohepatitis, cirrhosis, and fibrosis during a certain follow-up period of post-transplantation (37). Interestingly, the results of these studies, which are methodologically similar, vary significantly. This may be partly explained by the different follow-up durations between studies. Other explanations may include the difference in the study population, diagnosis criteria, and clinical features (38). Steatosis is common among the patients undergoing liver transplantation. Previous studies showed that steatosis mostly occurs in all the patients after five years of transplantation; however, fibrosis or cirrhosis seems to be rare (18). According to most previous studies, although there is a high incidence of recurrent NAFLD among the LT cases, steatosis mostly does not lead to graft rejection. In general, five year of prognosis among NAFLD patients undergoing LT is excellent (9, 10). 

On the other hand, the components of metabolic syndrome seem to be the main causes of recurrent NAFLD among LT patients. These components include obesity, insulin resistance, type 2 diabetes mellitus, arterial hypertension, and dyslipidemia. Andrade et al. (39) in a retrospective study aimed to estimate the prevalence ratio of recurrent and de novo NAFLD in LT cases (39). She found out that 56% of the patients with post- transplantation NAFLD belonged to the recurrent group. Narayaran et al. (40) in a prospective study with 254 LT cases compared the recurrent NAFLD and de novo incidences after two months of transplantation. In contrast to the previous study, they revealed that only 15% of the study population were suffering from recurrent NAFLD. Most of the post- transplantation NAFLD cases belonged to de novo arm. 

The other interesting study evaluated these two arms for longer period after transplantation. After a five-year follow-up, the prevalence of steatohepatitis seemed to be more significant in recurrent NAFLD, in comparison to de novo cases. Furthermore, the DM2 incidence was higher in the recurrent arm (41). This study concluded that recurrent NAFLD, in comparison to the de novo cases, is more severe and inevitable (41, 42). On the other hand, the need for a re-transplantation is lower in recurrent NAFLD patients, in comparison to the de novo NAFLD (42).

**Pediatric NAFLD following liver transplantation: **Pediatric NAFLD following LT has not been extensively studied. This is mostly due to the insufficient number of liver transplant cases. Nevertheless, there are few case reports of such patients, in which the indications of transplantation were some rare cases of NASH due to metabolic syndrome, and slightly more common were instances of progressive intrahepatic familial cholestasis type 1 (PFIC1) (43). The patients in these studies did not meet the criteria for metabolic syndrome.

**Risk factors associated with post-transplant NAFLD occurrence: **NAFLD affects both children and adults, yet the risk factors are mostly the same with a few differences mostly in the prevalence of etiologies. The following will discuss the risk factors of NAFLD, both in general and post-LT (44). Other risk factors include obesity, age, diabetes and metabolic syndrome in general, all of which traced back to an unhealthy lifestyle (45, 46). 

**Pre-transplant disease status: **The pre-transplant status of the patient is considered as an important risk factor for the occurrence of NAFLD following liver transplant (47). NAFLD is reported to be more common in liver transplant patients because of pre-existing NASH rather than other conditions (48). This is in part due to the lifestyle and other risk factors related to NASH in the first place (e.g. hypercholestrolemia, hypertension and diabetes mellitus). Nearly one-third of cirrhotic patients due to NASH cirrhosis are at increased risk of de novo NAFLD. Studies suggest that the NAFLD recurrence did not affect overall graft and/or patient survival up to 10 years (49). However, the risk of infection and cardiovascular-related morbidity and mortality appears to be more prevalent in these group of patients (49). 

The role of PNPLA3 rs738409-G allele as a risk factor for progression to NAFLD has been well-established. Recently, Finkenstedt et al. revealed that the presence of the rs738409-G allele among the recipients is considered to be an independent risk factor for the development of post-transplant steatosis (23). However, similar results were not observed among the liver donors with the same type of PNPLA3 polymorphism. Therefore, these results suggest that PNPLA3 rs738409-G allele is significantly associated with progression to post-transplant obesity and should be considered as an independent risk factor for de novo NAFLD (50, 51).

**Donor steatosis: **Donor steatosis is considered as a risk factor for NAFLD development, due to its role in graft survival, injury and rejection. The histological pattern of steatosis is also crucial, as a liver with macrovesicular steatosis is more susceptible to ischemia than a liver with microvesicular steatosis (52). Steatosis in the donor graft is a crucial predictor of transplant success, and several programs attempt to screen for steatotic donor livers accordingly. However, this may not be an accurate measure since a considerable number of mild cases of NAFLD neither display a change in blood aminotrasferase levels, nor present a clinical finding in ultrasonography (53-55). Invasive histological diagnosis is the only accurate and sensitive method for diagnosis of NAFLD. Nevertheless, it is not practically feasible due to the high number of donors. Moreover, it is worth noting that the predictive value of steatosis on graft survival is not supported by sufficient evidence. In fact, there are contradictory results from various studies, and some of them even highlight beneficial effects of steatosis on hepatic regeneration, albeit in animal models (56, 57). Interestingly, there are methods to “precondition” the donor liver to improve the quality and success rate of transplantation. Such methods include a specific diet, or in case of deceased donor organs, other techniques such as ischemic preconditioning (58, 59).

**Drugs: **In all organ transplant procedures, most patients receive immunosuppressive drugs such as corticosteroids and cyclophosphamides to prevent graft rejection (60). As these drugs can cause metabolic syndrome, they should also be considered as a risk factor for post-transplant NAFLD. Furthermore, these patients receive calcineurin inhibitors post-transplant, which additionally exacerbate hypertension and hypercholesterolemia. Other immunosuppressive drug regimens including tacrolimus and sirolimus are also diabetogenic and can disrupt various metabolism pathways. Moreover, cyclosporine and tacrolimus have significant adverse effects on human bone marrow. Both drugs are also associated with increased incidence of hyperlipidemia (61). The effects of tacrolimus and cyclosporine on graft survival and de novo diabetes have been compared in RCTs, favoring the use of tacrolimus (62).

Corticosteroids are usually a necessary element of the post-surgery drug regimen. They are also a major risk factor for post-surgical side effects. This is the result of corticosteroids' various effects on different parts of the body, which, in general, predispose the body in a metabolic-syndrome-like state. This phenomenon is known as the post-transplant metabolic syndrome, which increases the risk of cardiovascular events (63).

**Role of Angiotensin-converting-enzyme (ACE) inhibitors in NAFLD prevention: **A study revealed that the use of ACE inhibitors could be associated with decreased induction of de novo NAFLD post LT (odds ratio, 0.09; 95% conﬁdence interval, 0.01-0.92; P<0.042) (36). The mechanism is unclear, but it could be possibly related to the role of ACE-inhibitors in improving insulin sensitivity. On a molecular level, ACE-inhibitors appear to play a part in activation of peroxisome proliferator-activated receptor-γ, which is beneficial in NAFLD patients (64). Furthermore, ACE-inhibitors are antihypertensive, which reduce oxidative stress and hepatic fat accumulation. The latter is demonstrated to be a protective factor in hepatic fibrogenesis (65). Thus, nearly all medications used after liver transplant could disrupt the regular body metabolism. Further, each of these medications uniquely puts the patient at increased risk of post-transplant NAFLD (lipid metabolism disruption, protein disruption, etc.) and other complications such as cardiovascular events (66).

**Management: **The evidence is lacking with regard to the optimal pharmacological therapy of NAFLD following LT. However, it seems that anti-lipid agents, anti-hypertension drugs, and hypoglycemic therapies could control post-transplant NAFLD by decreasing the prevalence of metabolic syndrome’s components (43). Weight reduction combined with dietary modifications has shown to significantly improved post-transplant patient’s survival outcome (67, 68). In addition, morbidly obese patients should be candidates for weight reduction surgery as a complimentary treatment to LT (69). Modification of lifestyle or bariatric surgery before the transplant is numbered as the best way to prevent post-transplant recurrence of NASH (70). It is worth mentioning that a subset of patients undergoing LT may end up gaining weight following the surgery, which could lead to a compromised metabolic and results in a new-onset, post-transplant diabetes mellitus type 2 (71). While weight gain occurring during the first months after the transplant is indicative of the liver returning to its normal function, the same cannot be mentioned about weight gain in the long term (34). ILTS consensus conference recommended at least 10% of weight loss as a suitable target. They revealed 5% of weight loss could lead to positive outcome on hepatic histology and more weight reduction could minimize the liver injury and fibrosis (72). Nutritional status of the patient is also of great importance, since malnutrition is reportedly a common finding in end stage liver disease patients as well as in those with post-transplant setting (73). 

Lifestyle modification has different components, including dietary habits, exercise, and limited fatty acid intake. Previous studies suggested that the Mediterranean diet, which contains fruit, vegetables, whole grains, nuts, olive oils, fish, and poultry instead of red meat, could reduce the risk of NASH progression. In addition, this type of diet contributes to decreasing cardiovascular incidents by 30%. This type of diet not only decreases insulin resistance without weight reduction, but also helps to reduce the risks of related malignancies (72). Reducing the fatty acid intake could contribute to the inhabitation of NASH progression (74). According to the ILTS consensus conference on NAFLD and liver transplantation, the limited drink of alcohol helps to control NAFLD. Unfortunately, there is limited data about the role of mild use of alcohol in recurrent NAFLD (72). Although it is the best strategy available so far, many of the risk factors are ultimately inevitable. This makes it absolutely challenging in preventing the condition and causes a high prevalence of NAFLD in LT patients. 

In conclusion NAFLD is a common and rapidly growing disease both in the population as a whole and the subset population of liver transplant recipients. There has been multiple risk factors identified to play a crucial role in the development of post-transplant NAFLD. Nonetheless, there is limited data about the long-term outcome of liver transplant in patients with recurrent NAFLD. Further studies should work on this lack. NAFLD could affect the prognosis of the post-transplant patients/graft survival and also affects multiple different organs such as the heart and kidneys. Therefore, efficient management and prevention of NAFLD is strongly encouraged. Although efforts are being made to manage the risk factors in liver transplant patients, improved medication regimen and prevention methods should be taken into account to reduce the morbidity and mortality rates caused by NAFLD.
